# Prognostic Factors for the Survival of Elderly Patients Who Were Hospitalized in the Medical Ward of Our Hospital in Japan

**DOI:** 10.3390/geriatrics2040032

**Published:** 2017-11-02

**Authors:** Shuichi Abe

**Affiliations:** Internal Medicine, Rehabilitation Oomiko Hospital, Oomiko19, Oohara-cho, Tokushima-City, Tokushima 770-8012, Japan; walkers_high@yahoo.co.jp; Tel.: +81-88-662-1014; Fax: +81-88-662-2275

**Keywords:** AUC, elderly, prognostic factor

## Abstract

It has been a long time since there were many elderly people in Japan. The medical care and costs for the elderly are enormous, and research to lower the mortality rate of the elderly is needed. We retrospectively investigated the prognostic factors for the survival of elderly patients who were hospitalized in the medical ward of our hospital. In total, 277 patients who were hospitalized between 1 January 2014 and 31 May 2017, were included in the retrospective study. Univariate and multivariate analyses of items (vital signs, laboratory data, and so on) were performed, and significant differences between the survival group and death group were subjected to receiver operating characteristic curve analysis. Serum urea nitrogen levels and serum albumin levels provided a relatively high area under the curve (AUC). However, there was no item for which AUC exceeded 0.70, and setting the cutoff value in this study was difficult. For treating the elderly, it is important to carefully evaluate each patient’s prognostic factors, including the demented state, renal function, and nutritional state; personalized treatment of each patient is also important.

## 1. Introduction

The number of Japanese deaths in 2016 was 1,377,748, an increase of 17,304 from 1,294,444 in the previous year, and the death rate (per 1000 people) was 10.5, which was higher than the previous year’s 10.3. The rising trend of this mortality rate is due to the increase in the proportion of elderly people whose mortality rate is higher than other age groups due to the progress of aging. There are some prognostic factors for the survival of elderly patients. For example, an accurate estimation of the length of life for older hospitalized persons may be made using clinical information available from the medical chart plus a brief interview with the patient or surrogate [1]. A prognostic index using specific items from the comprehensive geriatric assessment (CGA) [2] in a large population of older hospitalized adults [3], a statistical and clinical prognostic value related to the functional and nutritional changes due to an acute illness [4], or a multidimensional prognostic index calculated from information collected in a standardized CGA [2] accurately stratifies hospitalized elderly patients into groups at varying risk of mortality [5]. Because no study in Japan has directly reviewed the clinical outcome of all hospitalized patients in a general internal medicine ward, which does not differentiate by age or illness, we conducted research on patients in the medical care ward of our hospital, and evaluated the prognostic factors for the elderly. This study was conducted to investigate whether data at our hospital and research results are consistent with the results of research at other facilities reported so far, or whether new findings are obtained.

## 2. Materials and Methods

The study was carried out in our hospital, a private hospital for the elderly that has a medical treatment ward with 60 beds in addition to a rehabilitation ward and a nursing care ward. In our hospital, the medical ward allows the hospitalization of patients from facilities such as nursing homes for the elderly and from other hospitals for medical treatment.

This investigation was a retrospective study. All subjects gave their informed consent for inclusion before they participated in the study. The study was conducted in accordance with the Declaration of Helsinki, and the protocol was approved by the ethics committee of our hospital (approval number: 0125). In order to give the opportunity for information disclosure and consent withdrawal, data collected from ordinary clinical information obtained from ordinary clinical practice was announced on the bulletin board of the hospital.

**Figure 1:**
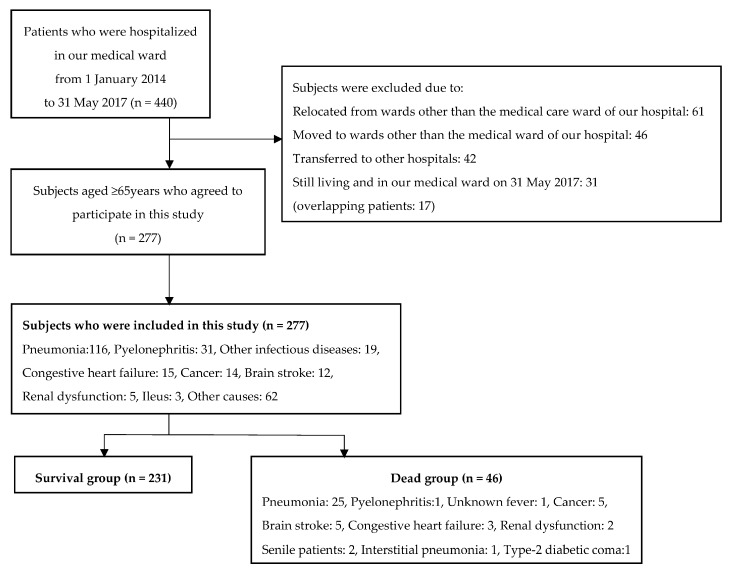
The flowchart of the study design.

Data was acquired from the paper charts of the hospital patients at our institution. Patients who were hospitalized in our medical ward from 1 January 2014 to 31 May 2017 were included in the study. The data collection date was 30 June 2017, and 440 patients were admitted to our medical ward during this period; All patients who were admitted to our medical ward were included in the study within the above mentioned date interval. Patients aged 65 years or older were registered in this study; 61 patients who relocated from wards other than the medical care ward of our hospital, 46 who moved to wards other than the medical ward of our hospital, 42 who transferred to other hospitals, and 31 who were still living and in our medical ward on 31 May 2017 were excluded from this analysis (17 overlapping patients). Therefore, in this study, 277 patients were enrolled (116 patients with pneumonia, 31 with pyelonephritis, 19 with other infectious diseases, 15 with congestive heart failure, 14 with cancer, 12 with brain stroke, five with renal dysfunction, three with ileus, and 62 with other causes). Forty-six patients died (25 with pneumonia, one with pyelonephritis, one with an unknown fever, five with cancer, five with a brain stroke, three with congestive heart failure, two with renal dysfunction, two senile patients, one with interstitial pneumonia, and one with a type-2 diabetic coma) (Figure 1). Patient background, underlying diseases, and laboratory findings were retrospectively analyzed. The activity of daily living level for each subject was rated in accordance with the “independence degree of daily living for the disabled elderly” (Table 1), which is used for assessments of the nursing care level needed under the long-term-care insurance system and is known to correlate closely with globally applied functional independence measures [6]. Dementia was also classified by the “independence degree of daily living for the demented elderly” (Table 2) to assess the daily assistance level needed under the long-term-care insurance system, which is known to correlate closely with mental state. Patients who died within 60 days after hospitalization were classified as the death group, and the other patients were classified as the survival group. Clinical factors for the survival group and death group were categorized by a history of bone fracture, cerebrovascular disorder, heart failure, malignant disease, presence or absence of steroids, sleeping pills, psychotropic drugs, and dementia treatment drug administration, and univariate analysis using the Fisher’s exact test was performed. Statistical analysis of three or more groups with ranking was conducted for the degree of independence in daily life. An analysis of numerical data such as age, white blood cell count, and C-reactive protein (CRP) level was statistically analyzed using the Mann-Whitney *U* test, and *p* < 0.05 was considered to be statistically significant. We also calculated the pairwise Pearson’s correlation coefficient and variance inflation factors to check multicollinearity. In the univariate and multivariate analyses, items with significant differences between the survival group and death group were subjected to receiver operating characteristic (ROC) analysis, and the area under the curve (AUC) value and cutoff value were analyzed. R version 3.4.0 (free software, 21 April 2017) was used for statistical analysis.

The geriatric nutritional risk index (GNRI) and controlling nutritional status (CONUT) are examples of indexes for evaluating nutritional status and were utilized in this study to determine their usefulness as prognostic indicators of survival in hospitalized elderly patients. GNRI was determined as 14.89 × serum albumin value (g/dL) + 41.7 × [current body weight (kg)/ideal body weight (kg)] to determine the risk of malnutrition (in cases where the current weight was over the ideal weight; the weight ratio was set to 1) [7]. The malnutrition risk was judged to be severe if GNRI was <82, moderate if it was >82 and <91, mild if it was >92 and <98, and no risk if it was >99. GNRI is an evaluation scale developed for elderly people aged ≥65 years, and it can be used for a prognostic prediction of survival of elderly stroke rehabilitation patients [8].

CONUT is an assessment tool to judge nutritional status from serum albumin values, total lymphocyte counts, and total cholesterol blood sampling results [9,10]. Because it does not require an examination or interview, it can be simply and objectively evaluated. In recent years, reports of CONUT use for patients with heart failure and hospitalization rehabilitation have been increasing. We used the CONUT score obtained by scoring the values of albumin, total cholesterol, and lymphocyte counts and accumulating the three scores as a nutritional index. The CONUT score is an indicator reflecting protein metabolism, lipid metabolism, and immunity, and the malnutrition level was evaluated in four stages: normal (CONUT score is 0–1), mild (2–4), moderate (5–8), and severe (9–12).

## 3. Results

Table 3 shows the background factors of the targeted cases. Among the 277 cases, 119 men (21 deaths) and 158 women (25 deaths), with a mean age of 84.9 ± 0.5 years (66–102 years) were registered. The hospitalization routes were 32 cases (four deaths) of hospitalization from home, 176 (26 deaths) of hospitalization from the facility, and 69 (16 deaths) of hospitalization from another hospital. The degrees of independence of daily life before hospitalization were 12 cases of J and A rank (0 deaths), 77 of B rank (four deaths), and 188 of C rank (42 deaths). The levels of dementia before hospitalization were 39 cases of level I (0 deaths), 41 of level II (three deaths), 82 of level III (nine deaths), and 115 of level IV to M (34 deaths).

The results of univariate and multivariate analyses are shown in Table 3 and Table 4. In Table 3, the reference category of sex is female, reference category of independence degree of daily living for the disabled elderly is level C, and reference category of independence degree of daily living for the demented elderly is level IV/M. Significant differences were found in the degree of dementia, age, body weight, GNRI, CONUT, body weight, body mass index, hospitalization day, maximum body temperature, oxygen saturation level in room air, oxygen flow rate after hospitalization, serum urea nitrogen value, serum creatinine value, serum sodium value, serum albumin value, serum cholinesterase value, and presence or absence of past stroke. White blood cell count and the serum CRP value had no effect on prognosis. The results of the multivariate analysis are shown in Table 5. Significant differences were found in age, serum urea nitrogen value, serum albumin value, and the degree of dementia.

In the univariate analysis in this study, gender was not involved in the prognostic factors. With regard to age, a significant difference was observed in vital prognosis in multivariate analysis. This suggests that chronological age plays an important role, as does biological age and physical fitness, and the passage of chronological age increases the risk of death and disease in the elderly.

Poor prognosis for malignant tumors, cerebral vessels, and cardiovascular disease complications has been reported in respiratory diseases [11], and our study showed significantly poor prognosis in cerebrovascular disorders. However, no significant difference was observed with respect to the white blood cell count and the CRP value, which were considered less useful for severity evaluation. In particular, CRP was not considered to be a prognostic factor.

In this study, the prognosis of the group with good self-reliance was significantly better, and a tendency for a poor prognosis in the low activity group was observed. This indicates the physical and functional status of the elderly as a prognostic factor.

AUC is defined by the ROC curve and is a tool for judging the usefulness of the prognostic factor [12]. In general, AUC is judged to be of high accuracy if it is ≥0.9, moderate accuracy if it is 0.7–0.9, and low accuracy if it is 0.5–0.7. The AUC of serum urea nitrogen levels in this study was 0.635, and AUC of serum albumin levels was 0.641; therefore, their predictive ability was not high. There was no item for which AUC was >0.70, and setting of the cutoff value in this study was difficult. Therefore, prognosis needs to be judged comprehensively for each patient.

## 4. Discussion

Generally, it is known that factors such as low activity state, anorexia, inflammatory response, and the like have great influences on life prognosis in elderly people with chronic diseases.

The older people’s mortality index was developed by four variables extracted from PCA (the principal components analysis), including BI (Barthel Index), age, hemoglobin, and mid-arm circumference [13]. Nutritional status is an indicator of the prognosis of the elderly [14]. Additionally, Hypoalbuminemia is an indicator of nutritional assessment and prognostic prediction [15]. Serum albumin levels decline with age, and the proportion of patients with hypoalbuminemia [16,17], nosocomial infections [18], commensal pneumonia [19], cancer and sepsis [20], and pressure sores [21] has been used as risk factors for death. In recent years, it has been reported that a low serum albumin level is a risk factor for ischemic heart disease [22] and cerebral infarction [23], which greatly influences the prognosis and quality of life of the elderly. Therefore, it is very important to maintain a good nutritional status.

Serum high-sensitivity CRP values at admission are predictors of short-term mortality at hospital admission in elderly multimorbid patients [24]. Inflammation seems to affect prognosis more than malnutrition in this setting and may therefore guide clinicians’ attitude towards therapeutic choices. The maximum body temperature on the hospitalization day was significantly lower in the death group. The exothermic reaction due to infectious diseases sometimes declines or disappears in the elderly [25]. This decline in exothermic reactions may delay the discovery of diseases such as infectious diseases in elderly people, which in turn leads to delayed treatment and may contribute to an increase in mortality. It is also thought that fever itself functions as a defense reaction against infection. Changes in exothermic reactions are dependent on qualitative and quantitative dysfunctions of inflammatory cytokines and are thought to be related to decreased immunity with aging [26,27]. Furthermore, it has been reported that a lack of chills is associated with poor prognosis in the elderly with pneumonia [28]. As with fever, the CRP value is considered to be dependent on inflammatory cytokines, but it was not found to be correlated with prognosis in this study. Based on these findings, it is considered important that the severity of disease in elderly people is not judged based on the strength of the inflammatory reaction and the degree of fever.

Severity assessment of dementia is reflected in the prognosis [29]. However, the nursing care provided to these residents should be guided by the goal of care, not estimating life expectancy.

In this study, we could use information obtained by our daily work and apply it to all patients regardless of patient background or illness. With regards to confounding factors, significant information was missing on the charts for variables such as the participants’ socioeconomic status and intervention of rehabilitation, and these could not be analyzed. The use of automated calculation processing via routine assessment by occupational staff other than physicians led to a decreased burden of this study work. Whether the results from this study will result in prognostic improvement is currently unknown and prospective cohort studies are needed in the future. In the study of the elderly prognostic factors in each country and each hospital, there are significant factors and there is a need to unify conditions and to accumulate and compare studies of elderly prognostic factors in each country and each hospital. Overall, for the treatment of the elderly, it is important to carefully evaluate each patient’s prognostic factors including demented state, renal function, and nutritional state, and in the actual clinical practice personalized treatment according to each elderly patient’s needs, the patient’s conditions and above data are also important because there are some differences in the importance of prognostic factors among past reports.

## Figures and Tables

**Table 1:** Independence degree of daily living for the disabled elderly.

**Self-supported**	**Rank J**	**Some disabilities, but daily living is mostly independent; capable of going outdoors unassisted.**
	1	Goes outdoors with means of transportation, etc.
	2	Goes out near home.
**Quasi-bedridden**	**Rank A**	**Indoor living predominantly independent, but unable to go out without assistance.**
	1	Goes out with assistance, spending most of the time during the daytime out of bed.
	2	Does not go out frequently, repeating cycles of lying down on and getting up from the bed during the daytime.
**Bedridden**	**Rank B**	**Some assistance needed for indoor living, also lies in bed for much of the daytime, although sitting position is possible.**
	1	Uses a wheelchair without assistance, takes meals, and excretes/urinates off the bed.
	2	Uses a wheelchair with assistance.
	**Rank C**	**Bedridden all day, requires assistance with excretion/urination, meals, and dressing/undressing.**
	1	Capable of changing posture in bed.
	2	Unable to change posture in bed without assistance.

**Note**: ADL was evaluated according to the scale for independence degree of daily living for the disabled elderly by the Japanese Ministry of health, labor, and Welfare based on interviews. **Abbreviation**: ADL, activities of daily living.

**Table 2:** Independence degree of daily living for the demented elderly.

**Rank I**	**Has some type of dementia, but almost independent in terms of daily living at home and in society.**
**Rank II**	**Some daily life-disturbing symptoms, behaviors and problems in communication seen but can lead daily life independently if kept watched by someone.**
IIa	Condition II, mentioned above, seen outside home.
IIb	Condition II, mentioned above, seen at home.
**Rank III**	**Daily life-disturbing symptoms, behaviors, and problems in communication that require assistance.**
IIIa	Condition III, mentioned above, seen primarily during the daytime.
IIIb	Condition III, mentioned above, seen primarily at night.
**Rank IV**	**Daily life-disturbing symptoms, behaviors, and problems in communication frequently require assistance.**
**Rank M**	**Marked psychiatric symptoms/related symptoms or serious physical disorders require expert management.**

**Note**: Demented level was evaluated according to the scale for independence degree of daily living for the demented elderly by the Japanese Ministry of health, labor, and Welfare based on interviews. Patients rated as rank III, IV, or M (having dementia requiring nursing care) was classified as “demented,” and all other patients were classified as “dementia-free”.

**Table 3:** Characteristics of the participants of this study (univariate analysis).

Variable	Survived (n = 231)	Died (n = 46)	Odds Ratio	95% Confidence Interval	*p*-Value
Sex			0.878	0.443–1.753	0.745
Male	98	21			
Female	133	25			
Hospitalization source					
Home	28	4			
nursing home	150	26			
other hospital	53	16			
Independence degree of daily living for the disabled elderly					0.200
JA	12	0			
B	73	4			
C	146	42			
Independence degree of daily living for the demented elderly					0.029 *
I	39	0			
II	38	3			
III	73	9			
IV/M	81	34			
History of bone fracture	97	17	0.810	0.394–1.624	0.623
History of brain stroke	117	15	0.473	0.224–0.959	0.0349 *
History of heart failure	80	20	1.450	0.719–2.891	0.313
History of malignant disease	46	14	1.755	0.798–3.721	0.120
Intake of steroid	15	3	1.005	0.179–3.775	1.000
Intake of sleeping pill	57	6	0.459	0.151–1.168	0.122
Intake of psychotropic drug	40	11	1.449	0.632–3.342	0.301
Intake of dementia treatment drug	42	5	0.550	0.160–1.551	0.285

* *p* < 0.05; reference category: sex (female), independence degree of daily living for the disabled elderly (level C), independence degree of daily living for the demented elderly (level IV/M).

**Table 4:** Relationship between numerical data and prognosis (univariate analysis).

Variable	Survived (n = 231)	Died (n = 46)	*p-*Value
Age (y.o.)	84.1 ± 8.6	88.8 ± 6.3	0.0001 *
Height (cm)	150.5 ± 10.2	148.1 ± 9.4	0.180
Weight (kg)	46.1 ± 9.6	42.0 ± 9.7	0.021 *
BMI (kg/m^2^)	20.4 ± 3.8	19.1 ± 3.8	0.048 *
The highest body temperature (°C)	37.4 ± 0.9	37.1 ± 0.8	0.038 *
Systolic blood pressure (mmHg)	133.3 ± 22.5	135.0 ± 23.5	0.718
Heart rate (beats/min)	81.0 ± 14.9	83.5 ± 21.7	0.607
Respiratory rate (times/min)	20.1 ± 6.5	21.5 ± 7.3	0.082
SpO_2_ (%)	93.2 ± 7.2	91.4 ± 5.0	0.002 *
Oxygen flow (L/min)	0.5 ± 1.1	1.5 ± 2.3	0.001 *
White blood Cell (×10^3^/μL)	8.6 ± 4.1	9.0 ± 4.2	0.445
Hematocrit (%)	33.8 ± 5.0	33.1 ± 5.0	0.282
Hemoglobin (g/dL)	11.5 ± 1.8	11.1 ± 1.7	0.235
Platelet (×10^4^/μL)	24.8 ± 12.6	22.5 ± 11.0	0.317
C-reactive protein (mg/dL)	4.2 ± 4.9	4.6 ± 5.2	0.217
Blood urea nitrogen (mg/dL)	19.3 ± 11.1	33.7 ± 34.7	0.012 *
Creatinine (mg/dL)	0.89 ± 0.54	1.24 ± 1.03	0.010 *
Serum sodium (mEq/L)	136.9 ± 5.1	135.7 ± 7.2	0.019 *
Serum potassium (mEq/L)	4.1 ± 0.7	4.3 ± 0.9	0.263
Total protein (g/dL)	6.5 ± 0.8	6.4 ± 0.8	0.555
Serum albumin (g/dL)	3.2 ± 0.6	2.9 ± 0.6	0.001 *
GNRI	84.5 ± 9.7	77.9 ± 10.5	0.0003 *
CONUT	4.8 ± 2.6	6.2 ± 2.6	0.002 *
Cholinesterase (IU/L)	190.4 ± 63.9	152.8 ± 60.7	0.0002 *
Total cholesterol (mg/dL)	161.4 ± 43.8	150.4 ± 51.3	0.085
Blood glucose (mg/dL)	128.5 ± 47.5	163.6 ± 203.4	0.370
HbA1c (NGSP) (%)	6.5 ± 10.3	5.9 ± 1.3	0.976

* *p* < 0.05; **Abbreviation**: NGSP, National Glycohemoglobin Standardization Program.

**Table 5:** Relationship between numerical data and prognosis (multivariate analysis).

Variable	Odds Ratio (Adjusted)	95% Confidence Interval (Adjusted)	*p*-Value
Age (y.o.)	1.09 (1.07)	1.04–1.15 (1.00–1.14)	0.027
Blood urea nitrogen (mg/dL)	1.04 (1.04)	1.02–1.06 (1.02–1.07)	<0.001
Serum albumin (g/dL)	0.37 (0.39)	0.21–0.66 (0.19–0.78)	0.006
Independence degree of daily living for the demented elderly	3.06 (2.76)	1.85–5.08 (1.63–4.68)	<0.001
